# Chloroform in Mania-a-potu

**Published:** 1849-06

**Authors:** 


*Chloroform in Mania-a-potu.*—Dr. G. Lane Corbin, of Virginia, writes to us that he has used chloroform successfully in two cases of mania-a-potu, after the various preparations of opium had failed to procure sleep.—*Medical News.*

